# CD8^+^ T Cells Form the Predominant Subset of NKG2A^+^ Cells in Human Lung Cancer

**DOI:** 10.3389/fimmu.2019.03002

**Published:** 2020-01-17

**Authors:** Yongyuan Chen, Zhongwei Xin, Lijian Huang, Lufeng Zhao, Shijie Wang, Jiwei Cheng, Pin Wu, Ying Chai

**Affiliations:** ^1^Department of Thoracic Surgery, Second Affiliated Hospital, Zhejiang University School of Medicine, Zhejiang University, Hangzhou, China; ^2^Cancer Institute, Second Affiliated Hospital, Zhejiang University School of Medicine, Zhejiang University, Hangzhou, China; ^3^Department of Thoracic Surgery, Henan Cancer Hospital, The Affiliated Cancer Hospital of Zhengzhou University, Zhengzhou, China; ^4^Key Laboratory of Tumor Microenvironment and Immune Therapy of Zhejiang Province, Second Affiliated Hospital, Zhejiang University School of Medicine, Zhejiang University, Hangzhou, China

**Keywords:** NKG2A, CD8^+^ T cells, non-small cell lung cancer, T-cell dysfunction, immune checkpoints, tumor microenvironment

## Abstract

**Background:** NKG2A is an inhibitory receptor of both T cells and natural killer (NK) cells. Persistent activation promotes T cells and NK cells to express NKG2A and results in the progression of chronic infection and cancer. However, the characteristics and subsets of NKG2A^+^ lymphocytes in human lung cancer are still unclear.

**Methods:** Here, we used the Tumor Immune Estimation Resource database and immune profiling of paired biospecimens to uncover the correlation between NKG2A expression and immune infiltration levels in human cancer as well as the characteristics of NKG2A^+^ lymphocytes in human lung cancer.

**Results:** We found that KLRC1 expression was especially correlated with CD8^+^ T-cell infiltration levels in 34 types of human cancer through the Tumor Immune Estimation Resource database. Moreover, NKG2A^+^ CD8^+^ T cells were the predominant subset of NKG2A^+^ lymphocytes in human lung cancer. In contrast, the NKG2A^+^ NK cells were decreased in tumors compared with the paired normal lung tissue. Tumor-infiltrating NKG2A^+^ CD8^+^ T cells expressed tissue-resident memory T cell (T_RM_ cell) and exhausted T-cell markers. Cytokines and cytotoxic molecules secreted by tumor-infiltrating NKG2A^+^ CD8^+^ T cells were significantly lower than those secreted by NKG2A^−^ CD8^+^ T cells *in vitro*. When stimulated with T-cell receptor activator, tumor-infiltrating NKG2A^+^ CD8^+^ T cells could secrete large amounts of granzyme B.

**Conclusions:** Our findings demonstrate that tumor-infiltrating NKG2A^+^ CD8^+^ T cells form the predominant subset of NKG2A^+^ cells in human lung cancer and suggest that targeting NKG2A^+^ CD8^+^ T cells is a promising approach for future anti-lung cancer immunotherapy.

## Introduction

Killer cell immunoglobulin-like receptors (KIRs) are a family of natural killer (NK) receptor that probe the proper expression of HLA class I on cells (1) involved in the control of cancer development and virus infection (2). According to their function, KIRs are classified into inhibitory receptors and stimulatory receptors (3). NKG2A is one of the inhibitory receptors in the KIRs family, which is expressed on both NK cells (4) and CD8^+^ T cells (5). NKG2A has been demonstrated to facilitate chronic infection through inhibiting the function of NK cells in various infectious diseases (5–8). However, the role of NKG2A in tumor immunity is less clear.

There are many similarities between the immune disorder of chronic infection and cancer (9). In terms of inhibitory receptors, the most famous similarity between chronic infection and cancer is the immune checkpoint (10, 11). A previous study showed that NKG2A promoted NK cell exhaustion and facilitated chronic hepatitis C virus infection in a mouse model (12). Recently, emerging evidence has demonstrated that the NKG2A blockade could promote both the NK and CD8^+^ T cell-mediated anti-tumor effect (13–15). Despite the remarkable progress in the understanding of the NKG2A blockade in tumor immunotherapy, there are many gaps in knowledge. For example, which is the dominant subset of NKG2A^+^ lymphocytes in human tumor tissue?

Here, we used the Tumor Immune Estimation Resource (TIMER) database to study the correlation between NKG2A expression and immune cell infiltration level in human cancer. In the TIMER database, we found that although the transcript level of NKG2A (KLRC1) in the tumor was lower than that in adjacent normal tissue, the expression level of KLRC1 was related to the immune infiltration levels in different types of cancers. Among the subsets of lymphocytes, the KLRC1 expression especially correlated with the CD8^+^ T-cell infiltration level in 34 types of human cancer. Therefore, we used immune profiling of paired peripheral blood (PB), tumor, and normal lung tissue to study the subsets of NKG2A^+^ lymphocytes and their characteristics in human lung cancer. We found that the quantity of NKG2A^+^ CD8^+^ T cells was much higher than that of NKG2A^+^ NK cells in human lung cancer.

Compared with paired PB and normal lung tissue, NKG2A expression was significantly increased on CD8^+^ T cells in tumors. In contrast, the NKG2A^+^ NK cells were decreased in tumors compared with that in paired normal lung tissue. NKG2A^+^ CD8^+^ T cells in tumors expressed tissue-resident memory T-cell (T_RM_ cell) marker CD103 and increased the expression of immune checkpoint PD-1. Cytokines and cytotoxic molecules secreted by tumor-infiltrating NKG2A^+^ CD8^+^ T cells were also significantly lower than those secreted by NKG2A^−^ CD8^+^ T cells *in vitro*. When reactivated by T-cell receptor (TCR) activator, tumor-infiltrating NKG2A^+^ CD8^+^ T cells increased the secretion of granzyme B, even with just a weak stimulus, but not IFN-γ. Our findings demonstrate that tumor-infiltrating NKG2A^+^ CD8^+^ T cells are the predominant subset of NKG2A^+^ cells in human non-small cell lung carcinoma (NSCLC) and suggest that targeting NKG2A^+^ CD8^+^ T cells is a promising approach for immunotherapy.

## Materials and Methods

### Online Database

The TIMER (cistrome.shinyapps.io/timer) database is a new website that involves 10,897 samples across 39 cancer types from The Cancer Genome Atlas (TCGA) for estimating the level of immune infiltration, and it provides six major analytic modules to deeply excavate molecular characterization of tumor-immune interactions including the Gene module, Survival module, Mutation module, SCNA module, Different expression module, and Correlation module. We first analyzed KLRC1 expression in different types of cancers by using the Different expression module. Then, we explored the clinical relevance of lung adenocarcinoma (LUAD) and lung squamous cell carcinoma (LUSC) via the Survival module. Next, we determined the correlation between the expression level of KLRC1and immune infiltration, including B cells, CD4^+^ T cells, CD8^+^ T cells, neutrophils, macrophages, and dendritic cells, via gene modules in diverse cancer types. Finally, the correlation module could be used to draw the scatterplots that represented the correlation between KLRC1 and CD8A expression in LUAD and LUSC. The x-axis and y-axis represent the expression level of KLRC1 and related marker genes, respectively. The gene expression level is converted into log2 RSEM. We analyzed the correlation between HLA-E expression and outcome of NSCLC in the OncoLnc database (http://www.oncolnc.org/).

### Tissue Collection

Tumor (T, homogeneous cellularity, without foci of necrosis), paired normal lung tissue (N), and some fresh PB were obtained from patients with NSCLC who underwent surgical resection at the Second Affiliated Hospital, Zhejiang University School of Medicine. Autologous PB was collected before surgery. Normal autologous tissue was obtained from a macroscopically normal part of the excised pulmonary lobe, at least 5 cm away from the tumor. None of the patients had received radiotherapy or chemotherapy before operation.

### Cell Preparations

Freshly excised tissues were cut into small pieces and then digested in RPMI 1640 medium containing 2% fetal bovine serum, type IV collagenase (1 mg/ml), and hyaluronidase (10 ng/ml) for 2–3 h at 37°C. PB lymphocytes were isolated after centrifugation on a Ficoll gradient.

### Antibodies and Flow Cytometry

The antibodies CD3 (UCHT1), CD8a (RPA-T8), CD103 (Ber-ACT8), CD279 (EH12.2H7), CD45 (HI30), CD56 (5.1H11), IFN-γ (B27), TNF-α (MAb11), granzyme B (QA16A02), HLA-E (3D12), and EpCAM (CO17-1A) were purchased from Biolegend, and NKG2A (REA110) was purchased from MiltenyiBiotec. We used mechanic dispersion and enzymatic digestion to prepare single cells of normal and tumor tissues for extracellular staining of immune markers. For blocking non-specific binding and staining with different combinations of fluorochrome-coupled antibodies, we pre-incubated fresh tissue cells (1 × 10^6^/ml) in a mixture of phosphate-buffered saline, 2% fetal calf serum, and 0.1% (w/v) sodium azide with FcgIII/IIR-specific antibody. Then, we followed the manufacturer's protocol after 12 h incubation in the presence of Leukocyte Activation Cocktail (BD Pharmingen) to perform intracellular staining. Fluorescence data were collected on a FACSCanto II system (BD Biosciences) and analyzed using FlowJo software (Tree Star).

### *In vitro* Culture

To investigate the cytokine secretion of tumor-infiltrating CD8^+^ T cells, single cells of normal and tumor tissues were cultured in the presence of Streptamer CD3/CD28 (Kit; Biolegend) or PMA and ionomycin. After a while, single cells of normal and tumor tissues were collected for the granzyme B, TNF-α, and IFN-γ assay.

### Immunofluorescence Staining

Paraffin-embedded and formalin-fixed samples were cut into 5-μm sections, which were then processed for immunofluorescent staining or immunohistochemistry staining. After incubation with antibodies against human CD8 and NKG2A, followed by Alexa Fluor 488- or 647-conjugated goat anti-mouse IgG or Alexa Fluor 488- or 649-conjugated goat anti-rabbit IgG (Invitrogen), images were acquired with a confocal microscope (Zeiss LSM 710, Carl Zeiss, Dublin, CA, USA).

### Statistical Analysis

Results are expressed as mean values ± SEM. Statistical analysis was performed by using GraphPad Prism software version 6.1. The statistical significance of differences between groups was determined by the Student's *t*-test. All data were analyzed using two-tailed tests unless otherwise specified, and we considered a *p* < 0.05 as statistically significant.

## Results

### Data Mining of the TIMER Database

**Figure 1:**
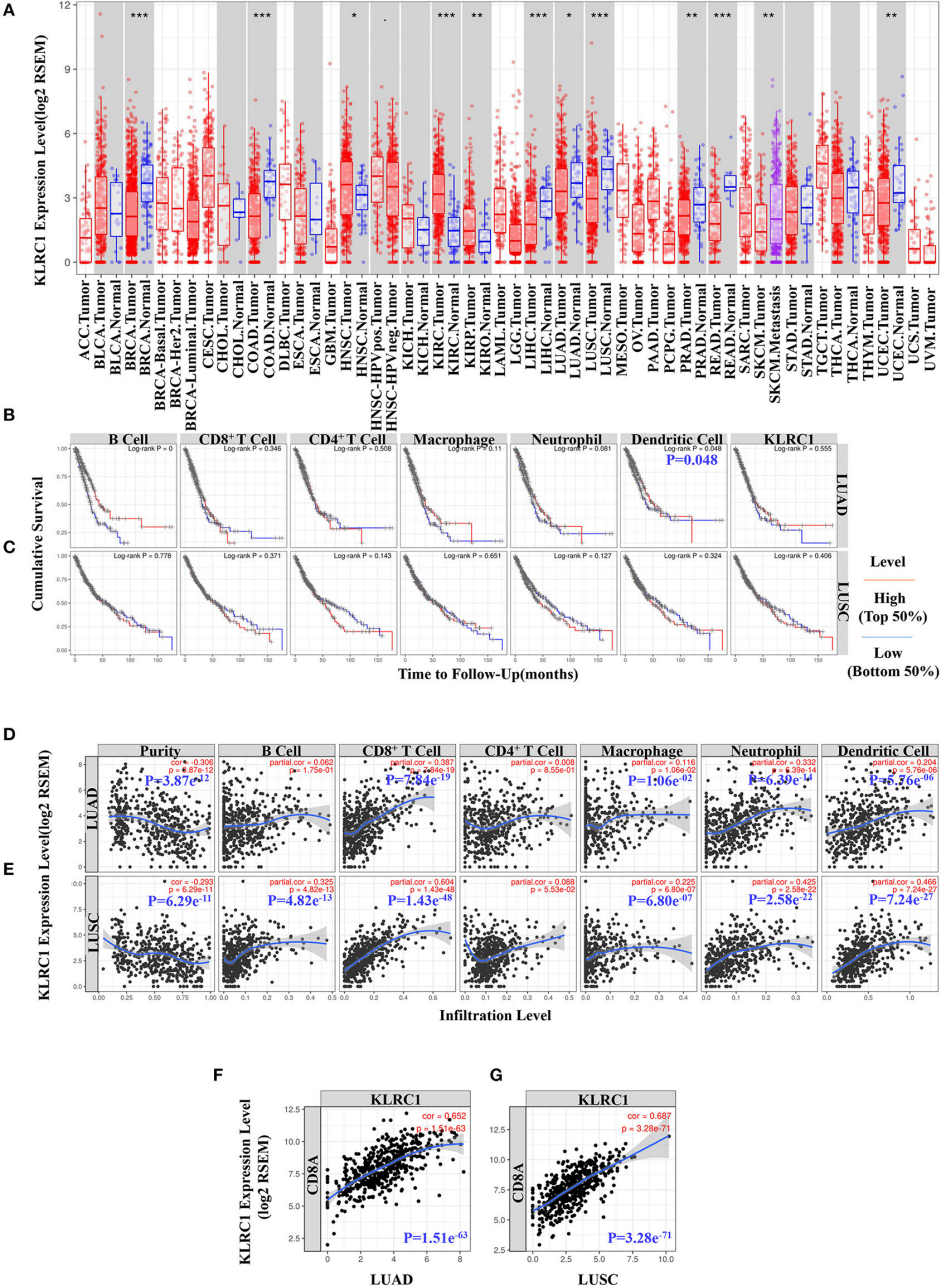
Data mining of the Tumor Immune Estimation Resource (TIMER) database. **(A)** Human *KLRC1* expression levels in different tumor types from the TCGA database were determined by TIMER (*p < 0.05, **p < 0.01, ***p < 0.001). **(B)** Prognostic roles of *KLRC1* and immune-related factors in lung adenocarcinoma (LUAD) from The Cancer Genome Atlas (TCGA) database were determined by TIMER. **(C)** Prognostic roles of *KLRC1* and immune-related factors in lung squamous cell carcinoma (LUSC) from the TCGA database were determined by TIMER. **(D)** *KLRC1* expression is significantly negatively related to tumor purity and has significant positive correlations with infiltrating levels of CD8^+^ T cells, macrophages, neutrophils, and dendritic cells in LUAD, but no significant correlations with infiltrating levels of B cells and CD4^+^ T cells. **(E)** *KLRC1* expression is significantly positively correlated with tumor purity and infiltrating levels of B cells, CD8^+^ T cells, macrophages, neutrophils, and dendritic cells in LUSC, other than CD4^+^ T cells. **(F)** Correlation between *KLRC1* and *CD8A* expression in LUAD. **(G)** Correlation between *KLRC1* and *CD8A* expression in LUSC.

To evaluate KLRC1 expression in human cancers, we used the RNA-seq data of multiple malignancies in TCGA to examine the expression level of KLRC1. The differences in the expression level of KLRC1 between the tumor and adjacent normal tissue in all TCGA tumors are shown in Figure 1A. KLRC1 expression was significantly lower in most of the tumors compared with adjacent normal tissues, such as breast invasive carcinoma, colon adenocarcinoma, liver hepatocellular carcinoma, LUAD, LUSC, PRAD, rectum adenocarcinoma, and uterine corpus endometrial carcinoma. However, the expression level of KLRC1 was significantly higher in head and neck cancer, kidney renal clear cell carcinoma, and kidney renal papillary cell carcinoma compared with adjacent normal tissues (Figure 1A).

In our study, we found that dendritic cells were correlated with the survival of LUAD (Figure 1B). However, there was no significant correlation between KLRC1 expression and survival of lung cancer (Figures 1B,C). Then, we investigated whether there was a correlation between KLRC1 expression and the immune infiltration levels in different types of cancers. We assessed the connection between KLRC1 expression and immune infiltration levels in 39 types of cancer through the TIMER database. In addition, we found that the expression of KLRC1 is negatively correlated with tumor purity in 32 tumor types and positively correlated with CD8^+^ T-cell infiltration levels in 34 tumor types. Moreover, KLRC1 expression has positive correlations with infiltrating levels of B cells in 23 cancer types, CD4^+^ T cells in 20 cancer types, macrophages in 17 cancer types, neutrophils in 31 cancer types, and dendritic cells in 32 cancer types (Figures 1D,E, and Supplementary Figure 1).

After that, we selected LUAD and LUSC, in which the expression level of KLRC1 has an obviously negative correlation with tumor purity in TIMER. It is interesting that the expression level of KLRC1 is highly correlated with immune infiltration in LUAD and LUSC. The KLRC1 expression level has significantly positive correlation with CD8^+^ T cells (*r* = 0.387, *P* = 7.84e^−19^), macrophages (*r* = 0.116, *P* = 1.06e^−02^), neutrophils (*r* = 0.332, *P* = 6.39e^−14^), and dendritic cells (*r* = 0.204, *P* = 5.76e^−06^) in LUAD (Figure 1D). Similarly, there are significantly positive correlations between the expression level of KLRC1 and infiltrating levels of B cells (*r* = 0.325, *P* = 4.82e^−13^), CD8^+^ T cells (*r* = 0.604, *P* = 1.43e^−48^), macrophages (*r* = 0.225, *P* = 6.80e^−07^), neutrophils (*r* = 0.425, *P* = 2.58e^−22^), and dendritic cells (*r* = 0.466, *P* = 7.24e^−27^) in LUSC (Figure 1E).

We also analyzed the correlation between KLRC1 expression and CD8A expression in LUAD and LUSC through the TIMER database. KLRC1 expression had a significantly positive correlation with CD8A in both LUAD (*r* = 0.652, *P* = 1.51e^−63^) and LUSC (*r* = 0.687, *P* = 3.28e^−71^) (Figures 1F,G). These findings suggest that KLRC1 is significantly positively correlated with immune infiltration in human lung cancer, especially CD8^+^ T cells.

### CD8^+^ T Cells Form the Predominant Subset of NKG2A^+^ Cells Infiltrated in Human Lung Cancer

**Figure 2:**
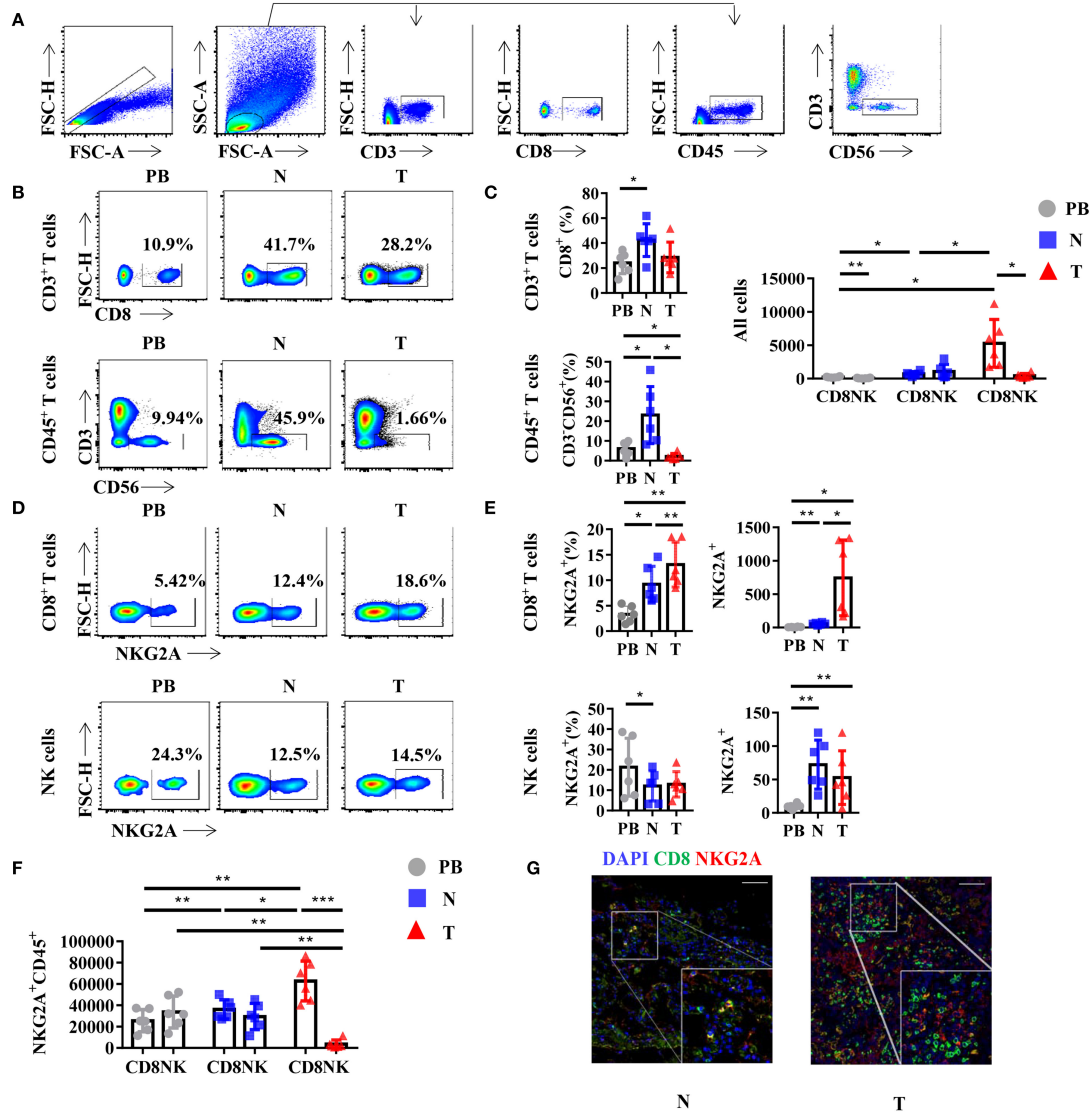
NKG2A expression on CD8^+^ T cells and natural killer (NK) cells in lung cancer. **(A)** Representative gating strategy for the flow cytometric analysis of CD8^+^ T cells and NK cells in NSCLC. **(B)** Representative flow cytometric analysis of CD8^+^ T cells (upper panels) in CD3^+^ leukocytes and NK cells (lower panels) in the CD45^+^ leukocytes in non-small cell lung carcinoma (NSCLC). Cells from peripheral blood (PB; *n* = 6), N (healthy normal tissue adjacent to the tumor, *n* = 6), and T (tumor, *n* = 6) were analyzed by flow cytometry. Numbers in plots indicate the percent of cells in respective gates. **(C)** Bar diagram shows the percentages of CD8^+^ T cells (upper panels) in CD3^+^ leukocytes and NK cells (lower panels) in the CD45^+^ leukocytes and absolute numbers in all the cells in NSCLC. Data are shown as the mean ± SEM; *n* = 6; *p < 0.05; **p < 0.01. **(D)** Representative flow cytometric analysis of NKG2A expression on CD8^+^ T cells (upper panels) and NK cells (lower panels) in NSCLC. **(E)** Bar diagram shows the NKG2A expression on CD8^+^ T cells (upper panels) and NK cells (lower panels) and its absolute numbers in all the cells in NSCLC. Data are shown as the mean ± SEM; *n* = 6; *p < 0.05; **p < 0.01. **(F)** Bar diagram shows the absolute numbers of CD8^+^ T cells and NK cells in NKG2A^+^ CD45^+^ leukocytes. Data are shown as the mean ± SEM; *n* = 6; *p < 0.05; **p < 0.01; ***p < 0.001. **(G)** Paraffin sections from lung cancer patients (scale bars represent 50 μm for right inserts) were stained with anti-human NKG2A (red) and anti-human CD8 (green) for immunofluorescent (IF) staining. One of six independent experiments is shown. N (healthy normal tissue adjacent to the tumor, *n* = 6) and T (tumor, *n* = 6).

Generally, NKG2A is recognized as an inhibitory receptor in the KIRs family that is mainly expressed on NK cells (2). Emerging evidence suggests that NKG2A also plays a crucial role in the anti-tumor immune response of CD8^+^ T cells (13, 14). However, whether CD8^+^ T cells or NK cells are the predominant subset of infiltrating NKG2A^+^ cells in human lung cancer is still unclear. Hence, we detected the percentage of NKG2A^+^ CD8^+^ T cells and NKG2A^+^ NK cells in the tumors of paired normal lung tissue and PB of human NSCLC by multi-color flow cytometry (Figure 2A). We found that the percentages of CD8^+^ T cells and NK cells were both decreased in tumors compared with that in paired lung tissue (Figures 2B,C). With regard to the quantity, the number of CD8^+^ T cells was significantly higher than that of NK cells in tumors (Figure 2C). In addition, we found that the percentage and absolute number of NKG2A^+^ CD8^+^ T cells were significantly increased in tumors compared with that in paired normal tissue and PB of NSCLC parents (Figures 2D,E). The percentage of NKG2A^+^ NK cells was lower in tumors and paired lung tissues than that in PB, but the absolute number of NKG2A^+^ NK cells was higher in tumors and paired lung tissues than that in PB (Figures 2D,E). More importantly, when we focused on the absolute numbers of NKG2A^+^ cells, we found that the number of tumor-infiltrating NKG2A^+^CD8^+^ T cells was significantly higher than that of NKG2A^+^ NK cells in tumors (Figure 2F). To further confirm the existence of NKG2A^+^ CD8^+^ T cells in tumors, we observed the co-localization of NKG2A and CD8 in tumors and normal lung tissues by immunofluorescence (Figure 2G). Our results suggest that NKG2A^+^ CD8^+^ T cells are the predominant subset of infiltrating NKG2A^+^ cells in NSCLC.

### CD103 and PD-1 Expression Is Increased on Tumor-Infiltrating NKG2A^+^ CD8^+^ T Cells

**Figure 3:**
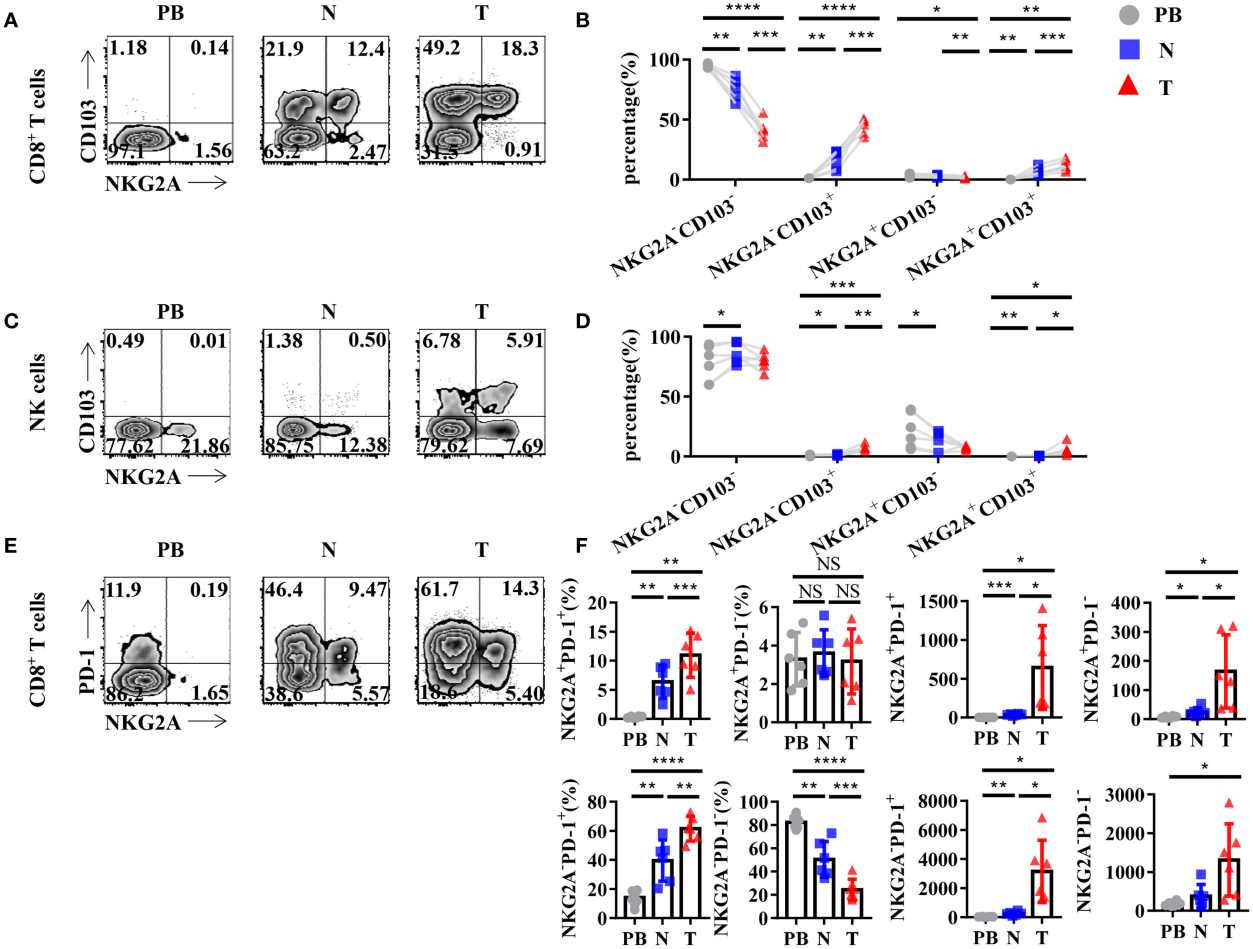
Tumor-infiltrating NKG2A^+^ CD8^+^ T cells express CD103 and PD-1. **(A)** Representative flow cytometric analysis of the expression of NKG2A and CD103 on CD8^+^ T cells in NSCLC. Cells from peripheral blood (PB; *n* = 6), N (healthy normal tissue adjacent to the tumor, *n* = 6), and T (tumor, *n* = 6) were analyzed by flow cytometry. **(B)** Bar diagram summarizes the expression of NKG2A and CD103 on CD8^+^ T cells in NSCLC. Data are shown as the mean ± SEM; *n* = 6; *p < 0.05; **p < 0.01; ***p < 0.001; ****p < 0.0001. **(C)** Representative flow cytometric analysis of the expression of NKG2A and CD103 on NK cells in NSCLC. **(D)** Bar diagram summarizes the expression of NKG2A and CD103 on NK cells in NSCLC. Data are shown as the mean ± SEM; *n* = 6; *p < 0.05; **p < 0.01; ***p < 0.001. **(E)** Representative flow cytometric analysis of the expression of NKG2A and PD-1 on CD8^+^ T cells in NSCLC. **(F)** Bar diagram summarizes the expression of NKG2A and PD-1 on CD8^+^ T cells in NSCLC and its absolute numbers. Data are shown as the mean ± SEM; *n* = 6; NS, no statistical significance; *p < 0.05; **p < 0.01; ***p < 0.001; ****p < 0.0001.

To further study the characteristics of NKG2A^+^ CD8^+^ T cells in NSCLC, we detected the expression of other surface markers on tumor-infiltrating NKG2A^+^ CD8^+^ T cells by flow cytometry. Interestingly, we found that tumor-infiltrating NKG2A^+^ CD8^+^ T cells expressed a high level of CD103 (95.3%) (Figure 3A), a marker of tissue-resident memory CD8^+^ T cells (T_RM_ cells) (16, 17). The percentage of NKG2A^+^ CD103^+^ CD8^+^ T cells was significantly increased in tumors compared with that in paired lung tissue and PB (Figure 3B). We also observed a similar trend of CD103 expression on tumor-infiltrating NKG2A^+^ NK cells (Figures 3C,D). Moreover, we found that the expression of PD-1, a marker of T-cell exhaustion (18), was increased in tumor-infiltrating NKG2A^+^ CD8^+^ T cells (72.6%) compared with that in paired lung tissue and PB (Figure 3E). The percentages of NKG2A^+^ PD-1^+^ CD8^+^ T cells and NKG2A^−^ PD-1^+^ CD8^+^ T cells were significantly increased in tumors compared with those in paired normal tissue and PB, but not NKG2A^+^ PD-1^−^ CD8^+^ T cells and NKG2A^−^ PD-1^−^ CD8^+^ T cells (Figure 3F). The absolute numbers of NKG2A^+^ PD-1^+^ CD8^+^ T cells and NKG2A^+^ PD-1^−^ CD8^+^ T cells were also higher in tumors than those in paired normal tissue and PB of NSCLC. These data suggest that human lung cancer-infiltrating NKG2A^+^ CD8^+^ T cells show the characteristics of both T_RM_ cells and exhausted T cells.

### Cytokine Profile of the Tumor-Infiltrating NKG2A^+^ CD8^+^ T Cells

**Figure 4:**
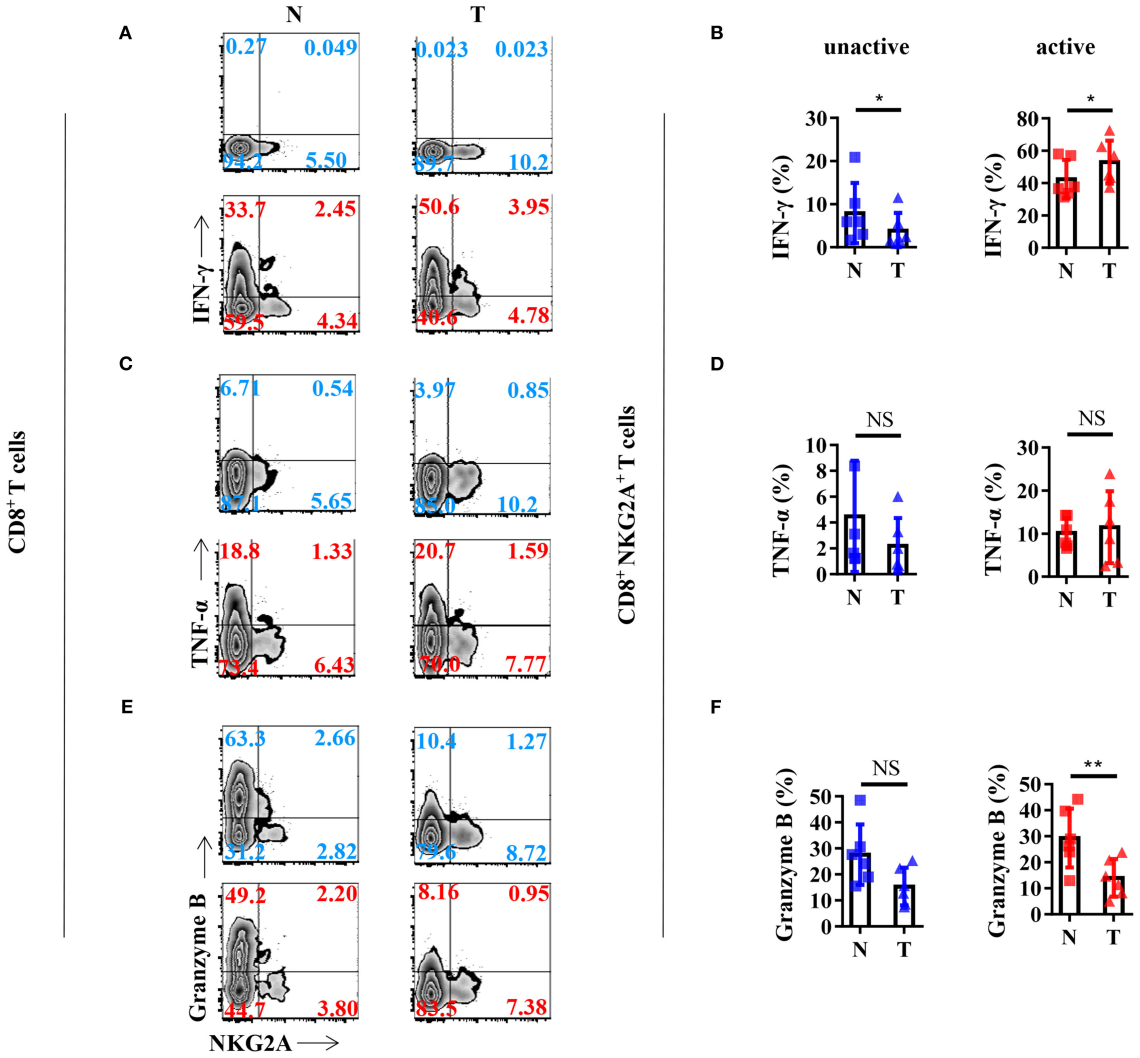
Anti-tumor cytokine profile of the NKG2A^+^ CD8^+^ T cells. **(A)** Representative flow cytometric analysis of the expression of NKG2A and intracellular IFN-γ produced by CD8^+^ T cells with BFA and Monensin (blue) or with BFA, Monensin, PMA, and Ionomycin (red) in NSCLC. **(B)** Cells from N (healthy normal tissue adjacent to the tumor, *n* = 6) and T (tumor, *n* = 6) were analyzed by flow cytometry. Bar diagram summarizes the levels of intracellular IFN-γ produced by CD8^+^ NKG2A^+^ T cells. Data are shown as the mean ± SEM; *n* = 6; *p < 0.05. **(C)** Representative flow cytometric analysis of the expression of NKG2A and intracellular TNF-α produced by CD8^+^ T cells. **(D)** Bar diagram summarizes the levels of intracellular TNF-α produced by CD8^+^ NKG2A^+^ T cells. Data are shown as the mean ± SEM; *n* = 6; NS, no statistical significance. **(E)** Representative flow cytometric analysis of the expression of NKG2A and intracellular granzyme B produced by CD8^+^ T cells. **(F)** Bar diagram summarizes the levels of intracellular granzyme B produced by CD8^+^ NKG2A^+^ T cells. Data are shown as mean ± SEM; *n* = 6; NS, no statistical significance; **p < 0.01.

In order to gain a deeper understanding of the biology of tumor-infiltrating NKG2A^+^ CD8^+^ T cells, we analyzed the cytokine profile of NKG2A^+^ CD8^+^ T cells from the tissues of NSCLC patients. We found that NKG2A^+^ CD8^+^ T cells from both tumors and paired normal tissue could be induced to express a high level of IFN-γ by *in vitro* activation through PMA and ionomycin (Figure 4A). The percentage of IFN-γ^+^ cells in tumor-infiltrating NKG2A^+^ CD8^+^ T cells was significantly higher than that in paired normal tissue upon *in vitro* activation (Figure 4B). However, the TNF-α expression of NKG2A^+^ CD8^+^ T cells was just slightly induced by *in vitro* activation (Figures 4C,D). Moreover, the percentage of granzyme B^+^ cells in tumor-infiltrating NKG2A^+^ CD8^+^ T cells was significantly decreased compared with that in paired normal tissue (Figures 4E,F). Interestingly, NKG2A^−^ CD8^+^ T cells secreted a large amount of granzyme B without activation in the normal tissue, but less in the tumor (Figure 4E). In contrast, after activation for 12 h, the percentage of granzyme B^+^ NKG2A^−^ CD8^+^ T cells from the tumor and normal tissue had a tendency to decrease, but not NKG2A^+^ CD8^+^ T cells (Figure 3E). These results suggest that tumor-infiltrating NKG2A^+^ CD8^+^ T cells have decreased anti-tumor potency, yet higher antitumor potential than NKG2A^−^ CD8^+^ T cells when activated.

### TCR Activation Promotes Tumor-Infiltrating CD8^+^ T Cells to Express NGK2A, PD-1, and Anti-tumor Cytokines

**Figure 5:**
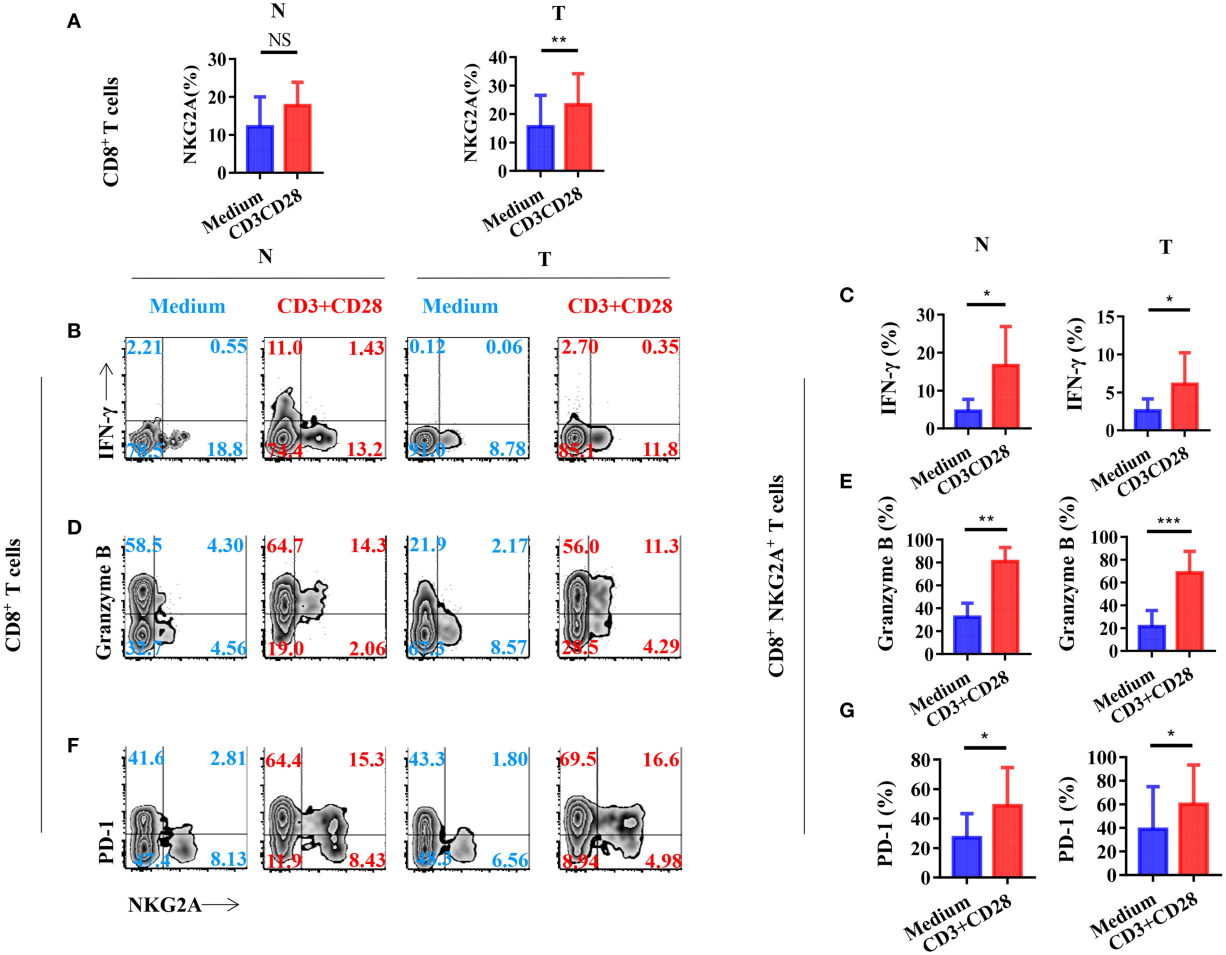
T-cell receptor (TCR) signal influences the expression of NGK2A and the secretion of IFN-γ and granzyme B. The single-cell suspension of paired normal tissue and tumor stimulated for 72 h with the recommended dose concentration of CD3 and CD28 or nothing. **(A)** Bar diagram shows the NKG2A expression change. N (healthy normal tissue adjacent to the tumor, *n* = 6), and T (tumor, *n* = 6). Data are shown as the mean ± SEM; *n* = 6; NS, no statistical significance; **p < 0.01. **(B)** Representative flow cytometric analysis of the expression of NKG2A and intracellular IFN-γ produced by CD8^+^ T cells. **(C)** Bar diagram summarizes the levels of intracellular IFN-γ produced by CD8^+^ NKG2A^+^ T cells. Data are shown as the mean ± SEM; *n* = 6; *p < 0.05. **(D)** Representative flow cytometric analysis of the expression of NKG2A and intracellular granzyme B produced by CD8^+^ T cells. **(E)** Bar diagram summarizes the levels of intracellular granzyme B produced by CD8^+^ NKG2A^+^ T cells. Data are shown as the mean ± SEM; *n* = 6; **p < 0.01; ***p < 0.001. **(F)** Representative flow cytometric analysis of the expression of NKG2A and PD-1 on CD8^+^ T cells. **(G)** Bar diagram summarizes the levels of PD-1 on CD8^+^ NKG2A^+^ T cells. Data are shown as the mean ± SEM; *n* = 6; *p < 0.05.

To investigate the mechanism of the generation of tumor-infiltrating NKG2A^+^ CD8^+^ T cells, we next stimulated CD8^+^ T cells *in vitro* with a TCR activator to determine the expression of NKG2A, PD-1, and cytokines. We found that the expression of NKG2A was significantly increased on tumor-infiltrating CD8^+^ T cells when activated *in vitro* (Figure 5A). TCR activation could induce the CD8^+^ NKG2A^+^ T cells derived from both the tumor and normal tissue to express IFN-γ (Figures 5B,C). Consistently, TCR activation could also induce CD8^+^ NKG2A^+^ T cells derived from both tumors and normal tissue to express granzyme B *in vitro* (Figures 5D,E). Moreover, TCR activation also significantly increased the expression of PD-1 on CD8^+^ NKG2A^+^ T cells derived from both tumors and normal tissue (Figures 5F,G). These findings illustrate that persistent activation of tumor-infiltrating CD8^+^ T cells could promote the expression of inhibitory receptors, such as NKG2A and PD-1, and facilitate tumor-infiltrating CD8^+^ T cells to become dysfunctional.

### The Expression of NGK2A on Tumor-Infiltrating CD8^+^ T Cells Is TCR Strength-Dependent

**Figure 6:**
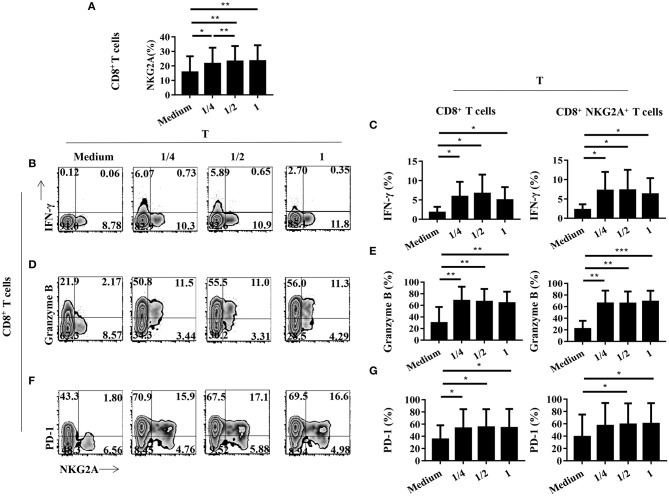
The expression of NGK2A on tumor-infiltrating CD8^+^ T cells is TCR strength-dependent. The single-cell suspension of paired normal tissue and tumor stimulated for 72 h with nothing or 1/4 or 1/2 or recommended dose concentration of CD3 and CD28. **(A)** Bar diagram shows the NKG2A expression change. N (healthy normal tissue adjacent to the tumor, *n* = 7) and T (tumor, *n* = 7). Data are shown as the mean ± SEM; *n* = 6; *p < 0.05; **p < 0.01. **(B)** Representative flow cytometric analysis of the expression of NKG2A and intracellular IFN-γ produced by CD8^+^ T cells. **(C)** Bar diagram summarizes the levels of intracellular IFN-γ produced by CD8^+^ T cells and CD8^+^NKG2A^+^ T cells. Data are shown as the mean ± SEM; *n* = 6; *p < 0.05. **(D)** Representative flow cytometric analysis of the expression of NKG2A and intracellular granzyme B produced by CD8^+^ T cells. **(E)** Bar diagram summarizes the levels of intracellular granzyme B produced by CD8^+^ T cells and CD8^+^NKG2A^+^ T cells. Data are shown as the mean ± SEM; *n* = 6; **p < 0.01; ***p < 0.001. **(F)** Representative flow cytometric analysis of the expression of NKG2A and PD-1in CD8^+^ T cells. **(G)** Bar diagram summarizes the levels of PD-1 in CD8^+^ T cells and CD8^+^NKG2A^+^ T cells. Data are shown as the mean ± SEM; *n* = 6; *p < 0.05.

We next studied the impact of TCR activation strength on the expression of NKG2A, PD-1, and cytokines on tumor-infiltrating CD8^+^ T cells *in vitro*. We found that the expression of NKG2A on tumor-infiltrating CD8^+^ T cells could be induced by weak TCR stimulation (Figure 6A). In addition, the expression of NKG2A on tumor-infiltrating CD8^+^ T cells was induced by weak TCR stimulation in a strength-dependent manner (Figure 6A). By contrast, the expression of PD-1 and cytokines on tumor-infiltrating CD8^+^ T cells did not show a significant tendency to associate with the strength of TCR activation in our study (Figures 6B–G). We further analyzed the expression of HLA-E, a ligand of NKG2A, in tumors and found that HLA-E was expressed on both tumor cells and immune cells in the tumors of NSCLC patients (Supplementary Figure 2). It was interesting that HLA-E expression was negatively correlated with the frequency of NKG2A^+^ CD8^+^ T cells in the tumor (Supplementary Figure 2). However, we did not observe a significant correlation between the transcriptional expression level of HLA-E and the outcome of NSCLC patients (Supplementary Figure 3). These findings indicate that NKG2A expression on tumor-infiltrating CD8^+^ T cells occurred in a TCR strength-dependent manner that was different from PD-1 expression. Our results also suggest that the accumulation of NKG2A^+^ CD8^+^ T cells in the tumor may be negatively regulated by HLA-E.

## Discussion

NKG2A is one of the inhibitory receptors in the KIRs family, which is well-demonstrated to play a crucial role in chronic infection. As NKG2A was originally identified on NK cells, most studies about NKG2A^+^ cells were focused on NK cells both in chronic infection and in cancer (4, 6, 15). However, the exact subsets of NKG2A^+^ cells in human lung cancer tissue were unclear. Here, we provide the first study to uncover the predominant subset of NKG2A^+^ cells in human lung cancer. Our results clearly demonstrate that tumor-infiltrating CD8^+^ T cells form the predominant subset of NKG2A^+^ cells in human lung cancer tissue but not NK cells. This finding is important because in the current understanding, receptors of the KIRs family are majorly connected with NK cell dysfunction, but not CD8^+^ T cells. In fact, our results show that both the proportion and number of NKG2A^+^ CD8^+^ T cells are significantly increased in human lung cancer. In contrast, the proportion and number of NKG2A^+^ NK cells in tumors were decreased. Therefore, our results suggest that NKG2A^+^ CD8^+^ T cells are the predominant subset of NKG2A^+^ lymphocytes in the human lung cancer microenvironment and need to be the focus of future basic biology and clinical immunological research.

Our study also elucidates that tumor-infiltrating NKG2A^+^ CD8^+^ T cells express a high level of CD103, a demonstrated marker of T_RM_ cells (19, 20). Increased CD103 expression facilitates T cells to reside in epithelial tissue via the interaction between CD103 and E-cadherin (21). Our results suggest that tumor-infiltrating NKG2A^+^ CD8^+^ T cells are a dysfunctional subset of tumor-associated T_RM_ cells in human lung cancer. It is also known that tumor-infiltrating NKG2A^+^ CD8^+^ T cells express a higher level of PD-1, a marker of T-cell exhaustion (18, 22). Together, these findings suggest that NKG2A^+^ CD8^+^ T cells are dysfunctional T cells that are long-term residents in human lung cancer tissue. Consistent with our study, there were two studies have shown that the NKG2A blockade could promote anti-tumor immunity by unleashing dysfunctional CD8^+^ T cells in tumors (13, 14). Despite the promising clinical prospects of the NKG2A blockade in cancer, the underlying details regarding NKG2A-mediated T-cell dysfunction in cancer are still unknown.

NKG2A expression on NK cells is well-demonstrated to be induced by cytokines, such as interleukin-21 (23). Previous studies showed that chronic stimulation with antigen or cytokines could also increases NKG2A expression on CD8^+^ T cells (24, 25). This evidence suggests that the underlying mechanism of NKG2A expression on CD8^+^ T cells is different from that on NK cells. Consistently, we observed an increasing tendency of NKG2A expression on CD8^+^ T cells isolated from tumors and paired normal tissue upon TCR stimulation *in vitro*. Moreover, we found that the increased level of NKG2A expression on CD8^+^ T cells is TCR-dependent. These findings indicate that NKG2A expression of CD8^+^ T cells in tumor is induced by prolonged TCR stimulation. In addition, previous studies have shown that increased NKG2A^+^ CD8^+^ T cells in the PB of NSCLC patients were correlated with tumor progression (26). We found that HLA-E, a ligand of NKG2A, is expressed on both tumor cells and immune cells in the tumors of NSCLC patients. Interestingly, HLA-E expression is negatively correlated with the frequency of NKG2A^+^ CD8^+^ T cells in the tumor, suggesting that the accumulation of NKG2A^+^ CD8^+^ T cells in the tumor microenvironment is regulated by HLA-E.

In conclusion, our results elucidate that tumor-infiltrating NKG2A^+^ CD8^+^ T cells form the predominant subset of NKG2A^+^ lymphocytes in human lung cancer but not NK cells. We also found that NKG2A^+^ CD8^+^ T cells in human lung cancer tissue are a novel tumor-infiltrating T-cell subset with unique characteristics. Moreover, we provide further evidence suggesting that the expression of NKG2A on tumor-infiltrating CD8^+^ T cells is TCR-dependent, which is different from that on NK cells. Our findings highlight that the NKG2A^+^ CD8^+^ T cell is a promising candidate for future basic research and clinical studies of cancer immunotherapy.

## Data Availability Statement

All datasets generated for this study are included in the article/Supplementary Material.

## Ethics Statement

All samples were anonymously coded in accordance with local ethical guidelines (as stipulated by the Declaration of Helsinki). The studies involving human participants were reviewed and approved by the Review Board of the Second Affiliated Hospital of Zhejiang University School of Medicine. The patients/participants provided their written informed consent to participate in this study.

## Author Contributions

YChe, ZX, LH, LZ, SW, and JC performed the experiments. YChe and ZX analyzed the data. YChe and PW designed the experiments, interpreted the data, and wrote the manuscript. PW and YCha supervised the project.

### Conflict of Interest

The authors declare that the research was conducted in the absence of any commercial or financial relationships that could be construed as a potential conflict of interest.
