# Supplementary material for: CD8+ T Cells Form the Predominant Subset of NKG2A+ Cells in Human Lung Cancer
Source: Front Immunol. 2020 Jan 17;10:3002. doi: 10.3389/fimmu.2019.03002 (PMC6979261; doi:10.3389/fimmu.2019.03002)
Supplement: Supplementary file 1 [file Data_Sheet_1.DOCX]

**SUPPLEMENTAL FIGURE LEGENDS**

**Supplementary Figure 1. Data mining of TIMER database**

A. KLRC1 expression is significantly negatively related to tumor purity and has significant positive correlations with infiltrating levels of CD8^+^ T cells, CD4^+^ T cells, macrophages, neutrophils and dendritic cells in ACC, but no significant correlation with infiltrating level of B cells.

B. KLRC1 expression is significantly negatively related to tumor purity and has significant positive correlations with infiltrating levels of CD8^+^ T cells, CD4^+^ T cells, neutrophils and dendritic cells in BLCA, but no significant correlation with infiltrating level of B cells and macrophages.

C. KLRC1 expression is significantly negatively related to tumor purity and has significant positive correlations with infiltrating levels of B cells, CD8^+^ T cells, CD4^+^ T cells, neutrophils, and dendritic cells in BRCA, but no significant correlation with infiltrating level of macrophages.

D. KLRC1 expression is significantly negatively related to tumor purity and has significant positive correlations with infiltrating levels of B cells, CD8^+^ T cells, CD4^+^ T cells, neutrophils, and dendritic cells in BRCA-Basal, but no significant correlation with infiltrating level of macrophages.

E. KLRC1 expression is significantly negatively related to tumor purity and has significant positive correlations with infiltrating levels of B cells, CD8^+^ T cells, CD4^+^ T cells, macrophages, neutrophils, and dendritic cells in BRCA-Luminal.

F. KLRC1 expression is significantly negatively related to tumor purity and has significant positive correlations with infiltrating levels of CD8^+^ T cells, CD4^+^ T cells, neutrophils and dendritic cells in BRCA-Her2, but no significant correlation with infiltrating level of B cells and macrophages.

G. KLRC1 expression is significantly negatively related to tumor purity and has significant positive correlations with infiltrating levels of CD8^+^ T cells, neutrophils and dendritic cells in CESC, but no significant correlation with infiltrating level of CD4^+^ T cells, B cells and macrophages.

H. KLRC1 expression is significantly negatively related to tumor purity and has significant positive correlations with infiltrating levels of CD8^+^ T cells in CHOL, but no significant correlation with infiltrating level of CD4^+^ T cells, B cells, macrophages, neutrophils and dendritic cells.

I. KLRC1 expression is significantly negatively related to tumor purity and has significant positive correlations with infiltrating levels of B cells, CD8^+^ T cells macrophages, neutrophils and dendritic cells in COAD, but no significant correlation with infiltrating level of CD4^+^ T cells.

J. KLRC1 expression is significantly negatively related to tumor purity and has no significant positive correlations with infiltrating levels of B cells, CD8^+^ T cells, CD4^+^ T cells, macrophages, neutrophils and dendritic cells in DLBC.

K. KLRC1 expression is significantly negatively related to tumor purity and has significant positive correlations with infiltrating levels of B cells, CD8^+^ T cells, CD4^+^ T cells, neutrophils and dendritic cells in ESCA, but no significant correlation with infiltrating level of macrophages.

L. KLRC1 expression is significantly negatively related to tumor purity and has no significant positive correlations with infiltrating levels of B cells, CD8^+^ T cells, CD4^+^ T cells, macrophages, neutrophils and dendritic cells in GBM.

M. KLRC1 expression is significantly negatively related to tumor purity and has significant positive correlations with infiltrating levels of B cells, CD8^+^ T cells, CD4^+^ T cells, macrophages, neutrophils, and dendritic cells in HNSC.

N. KLRC1 expression is significantly negatively related to tumor purity and has significant positive correlations with infiltrating levels of B cells, CD8^+^ T cells, neutrophils and dendritic cells in HNSC-HPVpos, but no significant correlation with infiltrating level of CD4^+^ T cells and macrophages.

O. KLRC1 expression is significantly negatively related to tumor purity and has significant positive correlations with infiltrating levels of B cells, CD8^+^ T cells, CD4^+^ T cells, macrophages, neutrophils, and dendritic cells in HNSC-HPVneg.

P. KLRC1 expression is no significantly negatively related to tumor purity and has no significant positive correlations with infiltrating levels of B cells, CD8^+^ T cells, CD4^+^ T cells, macrophages, neutrophils and dendritic cells in KICH.

Q. KLRC1 expression is significantly negatively related to tumor purity and has significant positive correlations with infiltrating levels of B cells, CD8^+^ T cells, neutrophils and dendritic cells in KIRC, but no significant correlation with infiltrating level of CD4^+^ T cells and macrophages.

R. KLRC1 expression is no significantly negatively related to tumor purity and has significant positive correlations with infiltrating levels of B cells, CD8^+^ T cells, CD4^+^ T cells, neutrophils and dendritic cells in KIRP, but no significant correlation with infiltrating level of macrophages.

S. KLRC1 expression is no significantly negatively related to tumor purity and has no significant positive correlations with infiltrating levels of B cells, CD8^+^ T cells, CD4^+^ T cells, macrophages and neutrophils in LGG, but significant correlation with infiltrating level of dendritic cells.

T. KLRC1 expression is significantly negatively related to tumor purity and has significant positive correlations with infiltrating levels of B cells, CD8^+^ T cells, macrophages, neutrophils and dendritic cells in LIHC, but no significant correlation with infiltrating level of CD4^+^ T cells.

U. KLRC1 expression is significantly negatively related to tumor purity and has significant positive correlations with infiltrating levels of CD8^+^ T cells and neutrophils in MESO, but no significant correlation with infiltrating level of B cells, CD4^+^ T cells, macrophages and dendritic cells.

V. KLRC1 expression is significantly negatively related to tumor purity and has significant positive correlations with infiltrating levels of B cells, CD8^+^ T cells, neutrophils and dendritic cells in OV, but no significant correlation with infiltrating level of CD4^+^ T cells and macrophages.

W. KLRC1 expression is no significantly negatively related to tumor purity but has significant positive correlations with infiltrating levels of B cells, CD8^+^ T cells, CD4^+^ T cells, macrophages, neutrophils and dendritic cells in PAAD.

X. KLRC1 expression is significantly negatively related to tumor purity and has significant positive correlations with infiltrating levels of CD8^+^ T cells, CD4^+^ T cells, and dendritic cells in PCPG, but no significant correlation with infiltrating level of B cells, macrophages and neutrophils.

Y. KLRC1 expression is significantly negatively related to tumor purity and has significant positive correlations with infiltrating levels of B cells, CD8^+^ T cells, CD4^+^ T cells, macrophages, neutrophils and dendritic cells in PRAD.

Z. KLRC1 expression is significantly negatively related to tumor purity and has significant positive correlations with infiltrating levels of CD8^+^ T cells and dendritic cells in READ, but no significant correlation with infiltrating level of B cells, CD4^+^ T cells, macrophages and neutrophils.

AA. KLRC1 expression is significantly negatively related to tumor purity and has significant positive correlations with infiltrating levels of B cells, CD8^+^ T cells, CD4^+^ T cells, macrophages, neutrophils and dendritic cells in SARC.

AB. KLRC1 expression is significantly negatively related to tumor purity and has significant positive correlations with infiltrating levels of B cells, CD8^+^ T cells, CD4^+^ T cells, macrophages, neutrophils and dendritic cells in SKCM.

AC. KLRC1 expression is significantly negatively related to tumor purity and has significant positive correlations with infiltrating levels of CD8^+^ T cells, neutrophils and dendritic cells in SKCM-Primary, but no significant correlation with infiltrating level of B cells, CD4^+^ T cells and macrophages.

AD. KLRC1 expression is significantly negatively related to tumor purity and has significant positive correlations with infiltrating levels of B cells, CD8^+^ T cells, CD4^+^ T cells, macrophages, neutrophils and dendritic cells in SKCM-Metastasis.

AE. KLRC1 expression is significantly negatively related to tumor purity and has significant positive correlations with infiltrating levels of CD8^+^ T cells, macrophages, neutrophils and dendritic cells in STAD, but no significant correlation with infiltrating level of B cells and CD4^+^ T cells.

AF. KLRC1 expression is significantly negatively related to tumor purity and has significant positive correlations with infiltrating levels of B cells, CD8^+^ T cells, neutrophils and dendritic cells in TGCT, but no significant correlation with infiltrating level of CD4^+^ T cells and macrophages.

AG. KLRC1 expression is no significantly negatively related to tumor purity and has significant positive correlations with infiltrating levels of B cells, CD8^+^ T cells, CD4^+^ T cells, macrophages, neutrophils and dendritic cells in THCA.

AH. KLRC1 expression is no significantly negatively related to tumor purity and has significant positive correlations with infiltrating levels of B cells, CD8^+^ T cells, CD4^+^ T cells, macrophages, neutrophils and dendritic cells in THYM.

AI. KLRC1 expression is significantly negatively related to tumor purity and has significant positive correlations with infiltrating levels of B cells, CD8^+^ T cells, CD4^+^ T cells, macrophages, neutrophils and dendritic cells in UCEC.

AJ. KLRC1 expression is significantly negatively related to tumor purity and has significant positive correlations with infiltrating levels of B cells, CD8^+^ T cells, CD4^+^ T cells, neutrophils and dendritic cells in UCS, but no significant correlation with infiltrating level of macrophages.

AK. KLRC1 expression is no significantly negatively related to tumor purity and has no significant positive correlations with infiltrating levels of B cells, CD8^+^ T cells, CD4^+^ T cells, macrophages, neutrophils and dendritic cells in UVM.

**Supplementary Figure 2. The analysis of NKG2A^+^ T cells and HLA-E^+^ cells in tumor**

A. Representative gating strategy for the flow cytometric analysis of CD8^+^ NKG2A^+^ T cells and Epcam^+^ HLA-E^+^ cells. Numbers in plots indicate the percentage of cells in respective gates.

B. Representative flow cytometric analysis of NKG2A^+^ T cells (upper panels) in CD8^+^ leukocytes, HLA-E^+^ cells and its Fluorescence Minus One (FMO) control (lower panels) in Epcam^+^ cells in NSCLC. Low: NKG2A^+^ T cell low group, n = 6. High: NKG2A^+^ T cell high group, n = 6.

C. Bar diagram shows the percentages of HLA-E^+^ cells in NSCLC. Data are shown as mean ± SEM; Low: NKG2A^+^ T cell low group, n = 6. High: NKG2A^+^ T cell high group, n = 6, *p < 0.05.

**Supplementary Figure 3. Data mining of OncoLnc database**

Prognostic roles of HLA-E in LUAD and LUSC, analysis in OncoLnc database.

1. Overall survival (OS) in patients with top 25% versus bottom 25% of HLA-E expression in LUAD.
2. OS in patients with top 25% versus bottom 25% of HLA-E expression in LUSC.
