# Supplementary figures and images for: CD8+ T Cells Form the Predominant Subset of NKG2A+ Cells in Human Lung Cancer
Source: Front Immunol. 2020 Jan 17;10:3002. doi: 10.3389/fimmu.2019.03002 (PMC6979261; doi:10.3389/fimmu.2019.03002)

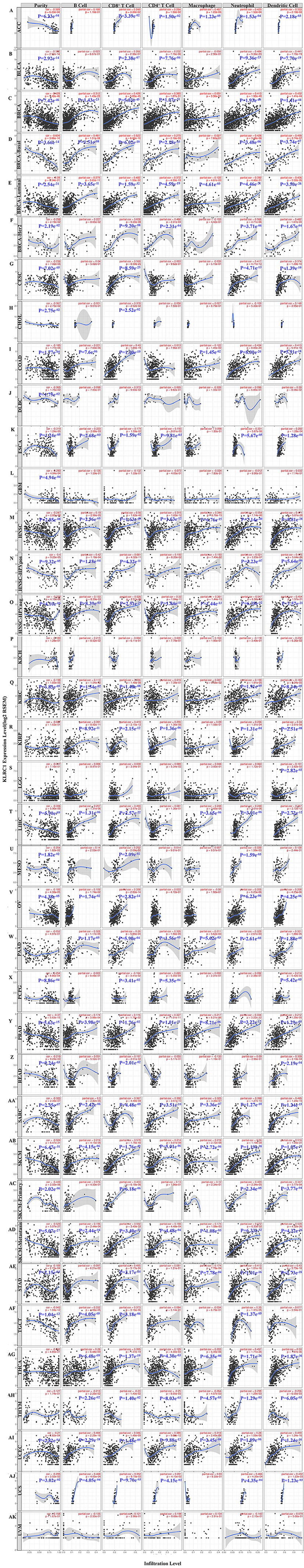

Supplement: Supplementary file 2 [file Image_1.jpeg]

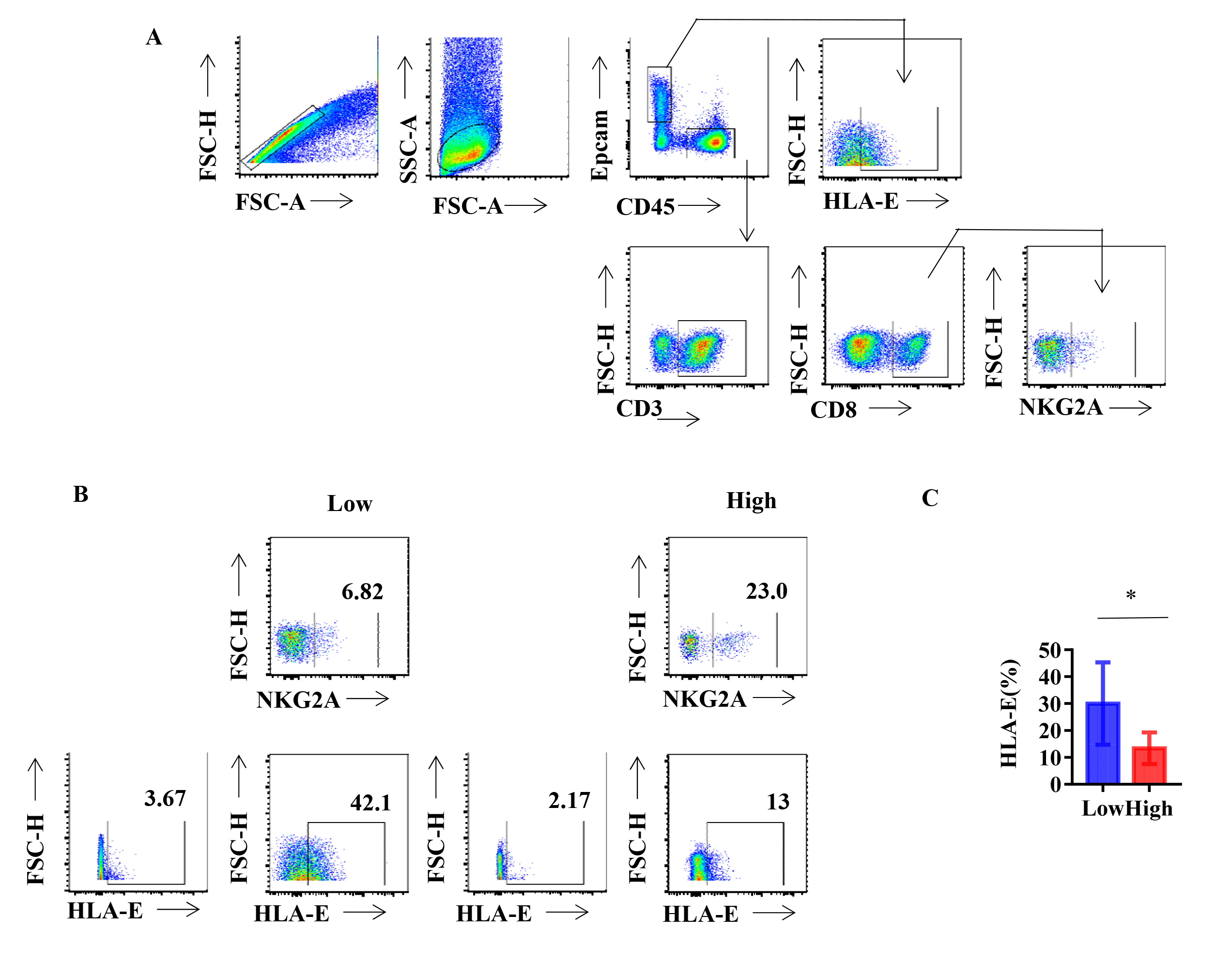

Supplement: Supplementary file 3 [file Image_2.TIF]

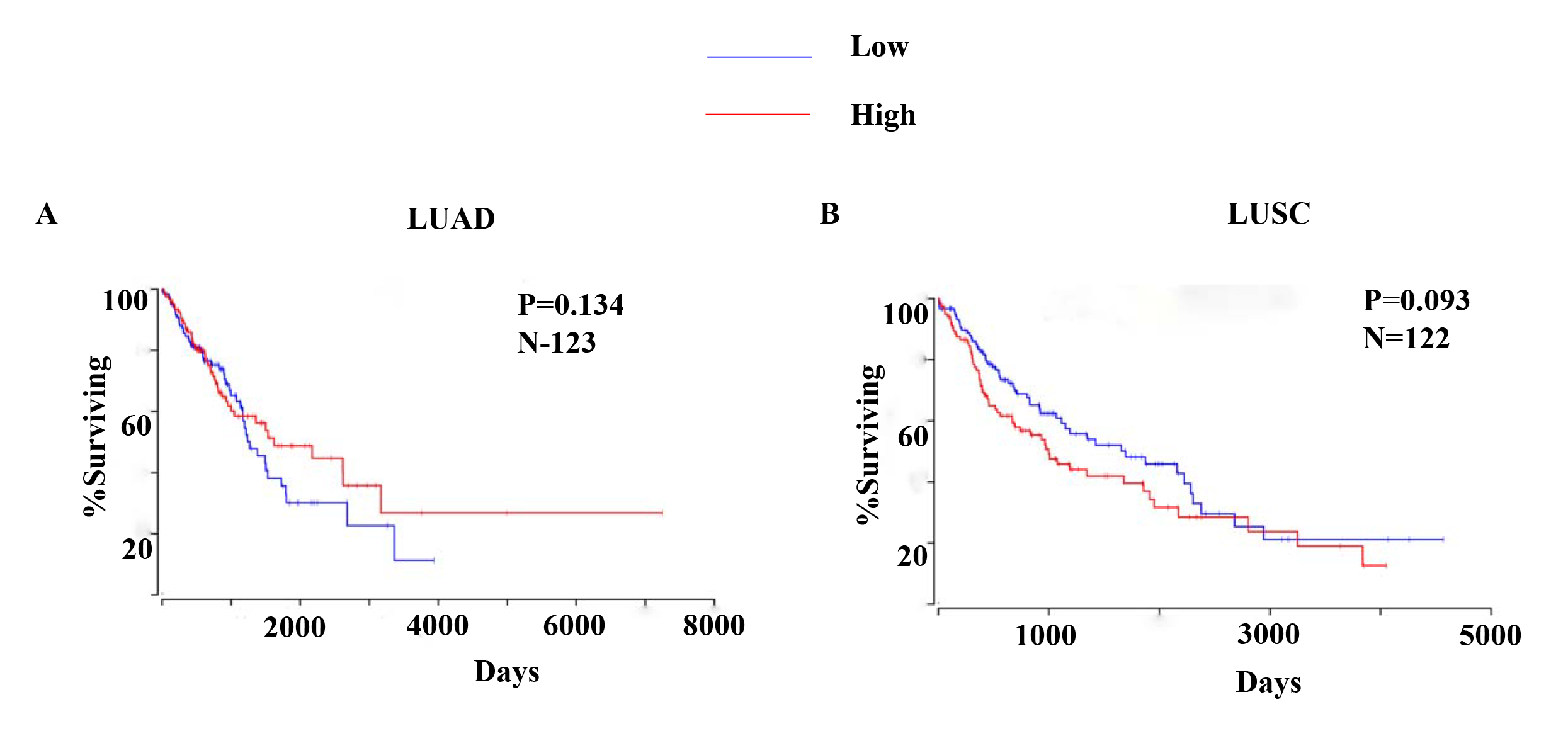

Supplement: Supplementary file 4 [file Image_3.TIF]
